# Incorporation of recombinant proteins into extracellular vesicles by *Lactococcus cremoris*

**DOI:** 10.1038/s41598-025-86492-z

**Published:** 2025-01-13

**Authors:** Tina Vida Plavec, Kristina Žagar Soderžnik, Giulia Della Pelle, Špela Zupančič, Robert Vidmar, Aleš Berlec

**Affiliations:** 1 Department of Biotechnology, Jožef Stefan Institute, Ljubljana, Slovenia; 2 Department for Nanostructured Materials, Jožef Stefan Institute, Ljubljana, Slovenia; 3 Department of Biochemistry and Molecular and Structural Biology, Jožef Stefan Institute, Ljubljana, Slovenia; 4 Faculty of Pharmacy, University of Ljubljana, Ljubljana, Slovenia

**Keywords:** Extracellular vesicles, *Lactococcus cremoris*, Recombinant protein, Delivery vehicle, Biotechnology, Microbiology, Molecular biology

## Abstract

Extracellular vesicles (EVs) are nanosized lipid bilayer particles released by various cellular organisms that carry an array of bioactive molecules. EVs have diagnostic potential, as they play a role in intercellular interspecies communication, and could be applied in drug delivery. In contrast to mammalian cell-derived EVs, the study of EVs from bacteria, particularly Gram-positive bacteria, received less research attention. This study aimed to investigate the production of EVs by lactic acid bacterium *Lactococcus cremoris* NZ9000 and to examine the impact of recombinant protein expression on their formation and protein content. Four different recombinant proteins were expressed in *L. cremoris* NZ9000, in different forms of expression and combinations, and the produced EVs were isolated using the standard ultracentrifugation method. The presence of vesicular structures (50–200 nm) in the samples was confirmed by transmission electron microscopy and by flow cytometry using membrane-specific stain. Mass spectrometry analyses confirmed the presence of recombinant proteins in the EVs fraction, with amounts ranging from 13.17 to 100%, highlighting their significant incorporation into the vesicles, together with intrinsic *L. cremoris* NZ9000 proteins that were either more abundant in the cytoplasm (ribosomal proteins, metabolic enzymes) or present in the membrane. The presence of the most abundant lactococcal proteins in EVs fraction suggests that protein cargo-loading of EVs in *L. cremoris* NZ9000 is not regulated. However, our data suggests that *L. cremoris* NZ9000 genetically engineered to express recombinant proteins can produce EVs containing these proteins in scalable manner. As *L. cremoris* NZ9000 is considered safe bacterium, EVs from *L. cremoris* NZ9000 could have several advantages over EVs from other bacteria, implying possible biotechnological applications, e.g. in therapeutic protein delivery.

**Supplementary Information:**

The online version contains supplementary material available at 10.1038/s41598-025-86492-z.

## Introduction

Extracellular vesicles (EVs) are nanosized lipid bilayer particles released by cells of various organisms, and involved in numerous cellular processes. They can carry diverse biological cargo, such as proteins, nucleic acids, and other molecules specific to the producing organism^1,2^. EVs play a crucial role in both intra- and inter-kingdom cellular communication, facilitating the transport and exchange of biological components^1,3^. Their significance in physiological processes, as well as their pathophysiological implications, has been widely acknowledged. Moreover, EVs isolated from body fluids possess diagnostic potential, as they may contain specific disease-related markers^4^. Their use as carriers of biomarkers for diagnostic purposes in clinical settings, such as in the initiation of inflammation and the development of cancer, has been demonstrated^5–8^.

Beyond their diagnostic potential and role in interspecies intercellular communication, EVs can be utilized for drug delivery^9,10^. They are recognized for their ability to transport biomolecules, making them promising next-generation delivery vehicles for therapeutics. Various cell types, including red blood cells^11^, mesenchymal stem cells^12^, and immune cells^13^, have been utilized as sources of EVs for drug delivery. Their use as delivery vectors for therapeutics offers numerous advantages. To start with, leveraging cellular machinery for drug loading helps prevent potential damage to sensitive biomolecules such as RNA and proteins during vesicle loading^14^. Vesicles are small and have phospholipid surface; properties which aid in evading the immune system^15,16^. EVs also possess the ability to transport cargo to different tissues, or even to remote areas of the body, and can traverse the blood-brain barrier, highlighting their exceptional bioactivity^17^. Furthermore, the recently explained concept of quantal secretion reinforces the efficacy of EVs as delivery systems, as it explains a potent impact on cells despite dilution of EVs at distant tissue^18^. Acting as carriers for bioactive compounds, EVs not only deliver the compound, but also shield the cargo from degradation during transit.

While extensive research has focused on mammalian cell-derived EVs, the exploration of bacterial EVs, particularly from Gram-positive bacteria, is more recent. Majority of the bacterial studies have focused on Gram-negative bacteria, where EVs are derived from outer membrane, and are therefore also termed outer membrane vesicles^19,20^. EVs from *Escherichia coli* have shown promise in immunotherapy of colon cancer^21,22^ and induced oxidative stress and mitophagy in cancer cells^21^. Additionally, attenuated strains of *Salmonella* Typhimurium have been explored as production platform for EV-based vaccines^23^.

However, EVs from Gram-negative bacteria pose potential toxicity risks due to the presence of lipopolysaccharides in the outer membrane. Also, attenuated Gram-negative strains could reacquire virulence. In contrast, EVs from Gram-positive bacteria have received less attention when it comes to EVs production, but show considerable promise^24–27^. *Lactococcus cremoris* has already emerged as a good alternative to *E. coli* in studying formation of intracellular vesicles and as an alternative host for the overexpression of membrane proteins through intracellular vesicles^28^. *L. cremoris* belongs to a group of lactic acid bacteria (LAB) which is renowned for its safe use in fermented food production, since is considered non-pathogenic. Furthermore, genetic engineering techniques for LAB are well-established and extensively used^29–32^, with LAB being recognized as potential drug delivery vehicles. In comparison to whole bacteria, EVs offer the advantage of smaller size and ability to reach distant organs. Moreover, the lipid bilayer structure of EVs may facilitate fusion with the membrane of human cells and internalization of the content, enabling more efficient therapeutic delivery. Some studies have already shown that EVs from LAB retain therapeutic benefits of the producer LAB, with EVs from probiotic *Lacticaseibacillus paracasei* having anti-inflammatory effects^33^ and EVs from *Lacticaseibacillus rhamnosus* and *Limosilactobacillus reuteri* positively modulating the immunity^27^. The objective of this study was to establish the ability of LAB *L. cremoris* NZ9000 to produce EVs, and test the influence of recombinant protein overexpression on their formation and protein content. For that purpose, four different recombinant proteins were expressed in *L. cremoris* NZ9000, namely binders of IL-6, IL-17R and IL-17 A (ZIL6, ARS019 and mFyn, respectively) with therapeutic potential^34,35^, as well as the fluorescent protein mCherry^36^. They were expressed in different forms and combinations, and the produced EVs were isolated using the standard ultracentrifugation method. Vesicular structures in the ultracentrifuged samples were confirmed with flow cytometry and transmission electron microscopy. Proteomic analyses enabled successful detection of all recombinant proteins, including two simultaneously expressed proteins, in the ultracentrifuged samples. Furthermore, by expressing red fluorescent protein in *L. cremoris* NZ9000, we generated fluorescent protein-carrying EVs, which could streamline EV analytics and visualization in further studies.

## Materials and methods

### Bacterial strains, growth conditions

*Lactococcus cremoris* NZ9000 ^37^ was grown at 30 °C in M17 medium (Merck, Darmstadt, Germany), supplemented with 0.5% glucose (GM-17) and chloramphenicol (10 µg/mL) for plasmid transformants, without agitation or in the same medium solidified with 1.5% agar. *L. cremoris* NZ9000 was transformed by electroporation^38^, using a Gene Pulser II apparatus (Bio-Rad, Hercules, CA, USA). The plasmids used in this study for transformation of *L. cremoris* NZ9000 are shown in Table 1.

**Table 1:** Plasmids used in the study.

Plasmid	Relevant features	References or source
pNZ8148	pSH71 derivative, PnisA, CmR, nisin-controlled expression	^39^
pSD-fZIL6	pNZ8148 containing gene fusion encoding Usp45 signal peptide (2.9 kDa), flag tag (1.0 kDa), ZIL6 (7.1 kDa), and cA (22.1 kDa)	^35^
pSD-fARS019	pNZ8148 containing gene fusion encoding Usp45 signal peptide (2.9 kDa), flag tag (1.0 kDa), ARS019 (5.7 kDa), and cA (22.1 kDa)	^34^
pNBBX	pNZ8148 containing NheI, BglII, BclI and XhoI restriction sites	^40^
p-mFyn	pNBBX containing gene fusion encoding Usp45 signal peptide (2.9 kDa), myc tag (1.2 kDa), fynomer (8.4 kDa) and cA (22.1 kDa)	^34^
p-fARS019-mFyn	pNBBX containing gene fusion encoding Usp45 signal peptide (2.9 kDa), flag tag (1.0 kDa), ARS019 (5.7 kDa), and cA (22.1 kDa) and gene fusion encoding Usp45 signal peptide (2.9 kDa), myc tag (1.2 kDa), fynomer (8.4 kDa) and cA (22.1 kDa)	^34^
pSC-fARS019	pNZ8148 containing gene fusion encoding Usp45 signal peptide (2.9 kDa), flag tag (1.0 kDa) and ARS019 (5.7 kDa)	^34^
p-Cher	pNZ8148 encoding mCherry (27.0 kDa)	^36^

* cA represents C-terminal anchoring domain of AcmA (cAcmA).

### Isolation of*** L. cremoris*** NZ9000-derived EVs

Expression of fusion proteins by engineered *L. cremoris* NZ9000 carrying different constructs was induced as previously described^34^. Briefly, overnight cultures of *L. cremoris* NZ9000 carrying different constructs were diluted (1:100) in 20 mL fresh GM-17 medium containing chloramphenicol (10 µg/mL) and grown to reach optical density at 600 nm (OD_600_) 0.8–1.0. Fusion protein expression was induced with 25 ng/mL of nisin (Fluka Chemie AG, Buchs, Switzerland). To prepare bacterial lysates, the cultures were incubated for another 3 h at 30 °C, followed by centrifuging the bacteria cultures at 4,500 × *g* for 20 min at 4 °C and resuspending the resulting pellet in 400 µL of PBS. For isolation of EVs, the cultures were incubated at 30 °C until they reached the OD_600_ value of 1.2–1.5 (approximately 1.5 h after inducing protein expression with nisin), as recommended previously^33,41^. The bacterial cells were pelleted by centrifugation at 4,500 × *g* for 20 min at 4 °C. Supernatant was passed through a 0.45-µm filter (Merck) to remove any remaining cells. Then, the filtrate was ultra-centrifuged at 130,000 × *g* for 2 h at 4 °C (Optima XPN-90, Beckman Coulter, Brea, CA, USA). Pellet containing EVs was resuspended in 100 µL of PBS, transferred to low-binding microcentrifuge tubes and stored at -80 °C for further experiments. The flow cytometry analysis was performed within 48 h of EVs preparation.

## Transmission electron microscopy

TEM was performed on isolated EVs using negative staining. EV sample was fixed for 30 min in 1% (v/v) glutaraldehyde in 0.008 M cacodylate buffer (pH 7.3) to match the osmolarity of PBS. 5 µL of fixed EV sample was transferred to formvar coated copper grids (200 mesh; Electron microscopy sciences, Hatfield, PA, USA) for 10 min, and excess liquid was gently removed with Whatman filter paper. Grids were briefly washed with milli-Q water by placing them on water drops, and excess liquid was removed. The grid was then placed over a drop of UA Zero (Agar Scientific, Stansted, UK) for 45 s, placed again on a water drop to remove the excess of stain, and subsequently air-dried. To observe the EVs structure and size, transmission electron microscopy (TEM) with energy-dispersive X-ray spectroscopy (EDS) was used (JEOL JEM-2100, Jeol Ltd., Tokyo, Japan).

## Polydispersity index and zeta potential analysis

Polydispersity index and zeta potential of the bacterial EVs containing selected recombinant proteins were measured using the dynamic light scattering technique. The measurements were carried out using a Malvern Zetasizer Nano ZS system (Malvern Panalytical Ltd., Malvern, UK). EV samples were diluted to 1 mL with PBS and measured at room temperature using “Liposomes” settings in polystyrene latex cuvettes (cell type: DTS0012).

## Flow cytometric analysis of EVs

The 100 µL sample containing EVs in PBS was divided into two microcentrifuge tubes equally. One was kept unstained and the other was stained with the fluorescent dye FM1-43 (0.5 µM, Thermo Fisher Scientific, Waltham, MA, USA) for 10 min at 37 °C. The samples were then diluted by adding 250 µL of filtered PBS and analyzed on Attune NxT Flow Cytometer (Thermo Fisher Scientific). To achieve the necessary resolution for visualizing EVs at the desired scale, Attune NxT small particle side-scatter filter (488/10) was used, at the lowest flow rate of 12.5 µL/min and the measurement limit of 50 µL. The following controls were included: unstained EVs, unstained and stained buffer, unstained and stained sterile growth medium. Triton X-100 was added at a final concentration of 1% (v/v) for 20 min to the stained samples containing EVs, and the samples were remeasured. FlowJo flow cytometry analysis software (version 10; Tree Star, Ashland, OR, USA) was applied for data analysis.

## Fluorescence intensity measurement

Expression of the mCherry protein in *L. cremoris* NZ9000/p-Cher was confirmed by measuring fluorescence intensity with excitation and emission maxima in the red region (587/610 nm) (Infinite M1000; Tecan, Männedorf, Switzerland). The measurements were performed in duplicates.

### Proteomic analysis of bacterial lysates and EVs by SDS-PAGE and Western blotting

The EV samples and bacterial lysates were thawed in an ice bath and briefly sonicated with a UPS200S sonicator (Hielscher Ultrasonics, Teltow, Germany). SDS-PAGE was performed with a Mini-Protean II apparatus (Bio-Rad) as previously described^34^. For silver staining, the gel was fixed in a fixing solution (50% (v/v) ethanol, 12% (v/v) acetic acid and 0.05% (v/v) formaldehyde (37%)) for 1 h. This was followed by washing in 50% (v/v) ethanol three times for 20 min. The gel was incubated in 0.02% (m/v) sodium thiosulfate, and rinsed with milli-Q purified water for 1 min. Then, the gel was incubated in 0.2% (m/v) silver nitrate with 0.05% (v/v) formaldehyde (37%) for 20 min, and rinsed with milli-Q purified water for 1 min. This was followed by incubating the gel in a developing solution (6% (m/v) sodium carbonate, 0.05% (v/v) formaldehyde (37%) and 0.2% (m/v) silver nitrate (2 mL per 100 mL)) until desired intensity of stain occurred. The reaction was stopped with washing in 50% (v/v) ethanol, 12% (v/v) acetic acid for 10 min, and the gel was washed in 50% (v/v) ethanol.

For Western blotting, the samples were mixed with 2x Laemmli Sample buffer and dithiothreitol (50 mM), and denatured by heating at 100 °C for 10 min before loading. Precision Plus Protein All Blue Standard (Bio-Rad) was used for molecular weight comparison. Proteins were transferred to a nitrocellulose membrane (GE Healthcare Life Sciences, Marlborough, MA, USA) using a semi-dry transfer with a protocol for 1.5 mm gels (Trans-Blot Turbo Blotting System; Bio-Rad). Membranes were blocked in 5% non-fat dried milk in TBST (50 mM Tris-HCl, 150 mM NaCl, 0.05% Tween 20, pH 7.5) and incubated overnight at 4 °C with flag tag rabbit polyclonal antibody (Proteintech, Rosemont, IL, USA; 1:1,000) and myc tag mouse polyclonal antibody (Proteintech; 1:1,000) in 5% non-fat dried milk in TBST. Following three washes with TBST, membranes were incubated for 2 h with secondary antibodies in 5% non-fat dried milk in TBST. For bacterial lysates samples, fluorescently labeled goat anti-rabbit IgG, StarBright 520 conjugate (Bio-Rad; 1:5,000) or goat anti-mouse IgG StarBright 700 (Bio-Rad; 1:5,000) were used. For EV samples, HRP conjugated secondary goat anti-rabbit IgG (1:5,000, Jackson ImmunoResearch, West Grove, PA, USA) or HRP conjugated secondary goat anti-mouse IgG (1:5,000, Jackson ImmunoResearch) were used to increase sensitivity. After three further washes with TBST, images were acquired using an imaging system (ChemiDoc MP, Bio-Rad). When using HRP labeled secondary antibodies, membranes were incubated with Clarity Max ECL substrate chemiluminescent reagent (Bio-Rad) prior to imaging.

## Proteomic analysis by mass spectrometry

The EV samples were sonicated as above to disrupt the EVs membrane and release the protein content. Bacterial cell lysates were also sonicated, and the soluble cell lysate of *L. cremoris* NZ9000 was included as a control. DC protein assay (Bio Rad) was used to estimate the protein concentration in the solution. Then, the samples were prepared for mass spectrometry analysis without pre-fractionation, according to the in-solution digestion protocol^42^, in which the proteins are first denatured, then the S-S bonds are reduced and protected by alkylation. This was followed by trypsinization and pre-purification of the peptides on the C18 carrier. LC-MS/MS analysis was performed with Orbitrap LTQ Velos mass spectrometer (Thermo Fisher Scientific) coupled to an EASY-nanoLC II HPLC (Thermo Fisher Scientific). The peptides were separated with a 90 min linear gradient of 5–50% solvent B (100% CAN in 0.1% FA) at a flow rate of 300 nL/min. MS mass spectra were recorded at a resolution of 30,000, with a mass range of 300–2000 m/z. The nine most intense ions from the full MS spectra were fragmented by HCD fragmentation. The MS/MS spectra were recorded at a resolution of 7500. Dynamic exclusion was enabled with a repeat count of 1, repeat duration of 30 s, and exclusion duration of 30 s. Protein identification from the recorded spectra was based on *L. cremoris* database (UniProt Archive, Proteome ID UP000007090), to which sequences of the recombinant proteins included in the study were manually added.

*L. cremoris* NZ9000 proteins that were identified with mass spectrometry in different samples were associated with their bioinformatic data available in Human Oral Microbiome Database (V3.1)^43^ and UniProtKB (Gene Ontology, Cellular component;^44^ (Supplementary data). Instead of the number of spectral counts (SC), normalized spectrum abundance factor (NSAF) score was calculated for all identified proteins to account for protein length and facilitate their relative quantification. The NSAF for an individual protein was obtained by dividing the number of SC with individual protein length (AA; number of amino acids, SC/AA for individual protein was then divided by the sum of SC/AA for all identified proteins in the sample, yielding spectrum abundance factor (SAF))^45^. Proteins identified in all samples were sorted according to the NSAF score of proteins in the cell lysate (calculated as SAF×100). The number of EV samples in which individual protein was identified (#Samples) was recorded as an indicator of abundance of this protein (intrinsic *L. cremoris* NZ9000 protein or expressed recombinant protein) in the vesicles in general. Proteins in vesicles were categorized on the basis of gene ontology descriptors into the following five classes: Ribosome (ribonucleoprotein complex [GO:1990904]; ribosome [GO:0005840]; large ribosomal subunit [GO:0015934]; small ribosomal subunit [GO:0015935]), Cytoplasm (cytoplasm [GO:0005737]), Membrane (plasma membrane [GO:0005886]; membrane [GO:0016020]), ABC transporter (ATP-binding cassette (ABC) transporter complex [GO:0043190]) and Other (other GO descriptors or not defined). For recombinant proteins, portion of total protein NSAF was calculated by dividing recombinant protein NSAF with the sum of NSAFs of all proteins identified in the sample.

## Results

### Isolation of EVs from genetically engineered ***L. cremoris*** NZ9000 carrying different plasmids and their visualization with electron microscopy

**Fig. 1:**
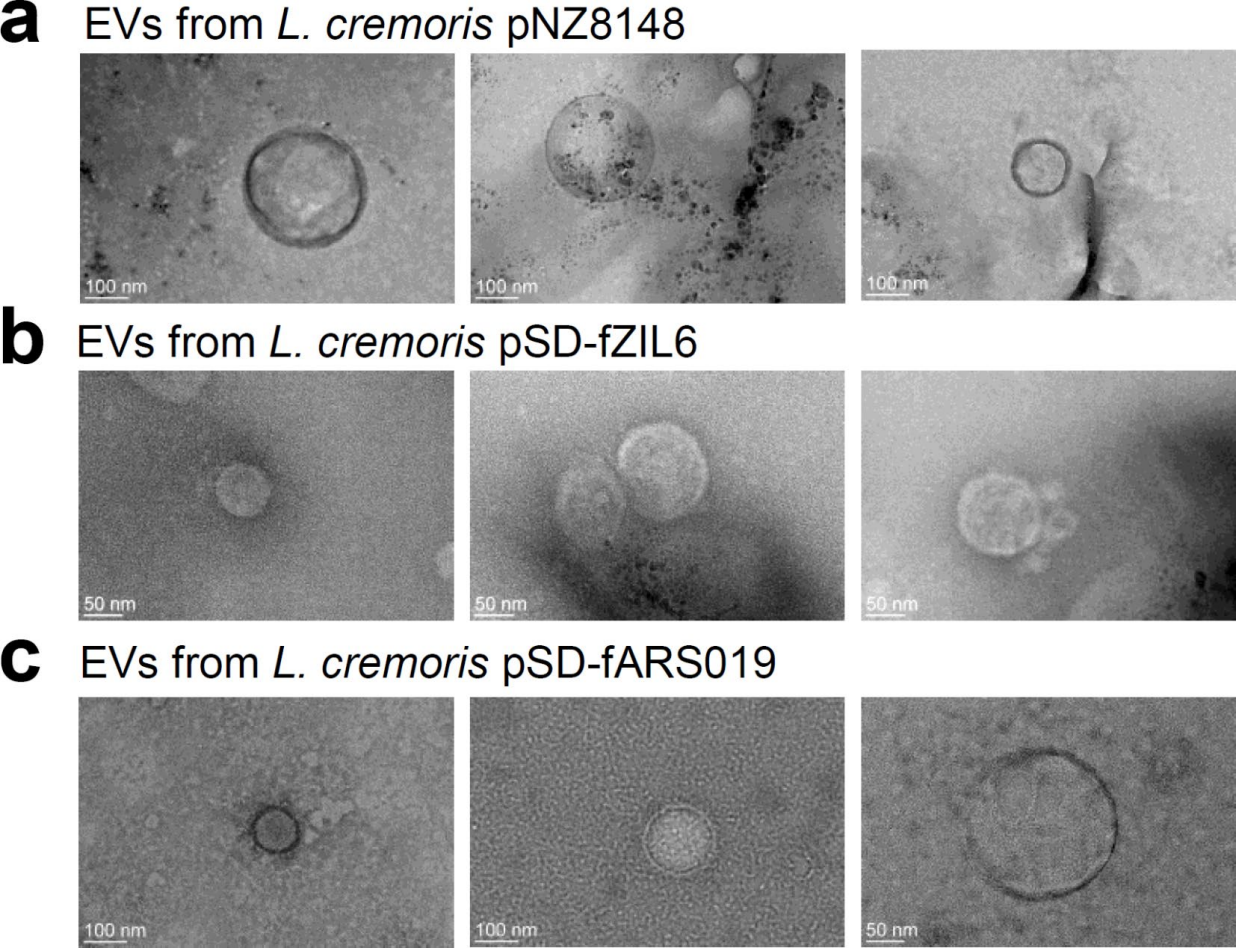
TEM analysis of *L. cremoris* NZ9000 ultracentrifugation samples reveals the presence of EVs. (**a**) : EVs from *L. cremoris* NZ9000/pNZ8148, (**b**): EVs from *L. cremoris* NZ9000/pSD-fZIL6, (**c**): EVs from *L. cremoris* NZ9000/pSD-fARS019. Scale bars indicate 50–100 nm.

*L. cremoris* NZ9000 harboring empty plasmid (*L. cremoris* NZ9000**/**pNZ8148) and plasmids encoding recombinant proteins ZIL-6 and ARS019 (*L. cremoris* NZ9000**/**pSD-fZIL6 and *L. cremoris* NZ9000**/**pSD-fARS019) were cultivated to early stationary phase and a standard EVs isolation protocol using an ultracentrifuge was followed as described in the Materials and methods. To confirm the presence of EVs in ultracentrifugation pellet, we performed transmission electron microscopy (TEM) with negative staining. Typical donut-shaped spherical structures of characteristic size (50–200 nm) and surrounded by lipid bilayer were observed in samples from selected recombinant *L. cremoris* NZ9000. Three representative images were selected for each of the EV samples (Fig. 1). In contrast, no vesicular structures were detected in the control samples made from sterile bacterial growth medium.

### Polydispersity and zeta potential of EVs isolated from genetically engineered *L. cremoris* NZ9000

EVs produced from *L. cremoris* NZ9000/pNZ8148 had a greater diversity in size compared to those from *L. cremoris* NZ9000/pSD-fZIL6 and pSD-fARS019, as revealed by measuring the polydispersity index (Table 2), which quantifies the uniformity of particle sizes in a sample. Lower polydispersity index values indicate greater uniformity, while higher values indicate greater heterogeneity^46^. EVs from *L. cremoris* NZ9000/pNZ8148 had approximately twice as high polydispersity index as EVs from *L. cremoris* NZ9000/pSD-fZIL6 and pSD-fARS019, signifying a higher level of heterogeneity. The zeta potential was found to be relatively high, suggesting weak charge of the EVs and higher propensity for their aggregation (Table 2).

**Table 2:** Polydispersity index and zeta potential of EVs from three selected recombinant *L. cremoris* NZ9000. Results are expressed in mean ± standard deviation from three measurements.

Bacterial strain	Polydispersity index	Zeta potential (mV)
*L. cremoris* NZ9000/pNZ8148	0.68 ± 0.29	-4.51 ± 1.19
*L. cremoris* NZ9000/pSD-fZIL6	0.38 ± 0.15	-4.88 ± 1.26
*L. cremoris* NZ9000/pSD-fARS019	0.25 ± 0.10	-5.34 ± 1.71

### Characterization of EVs with flow cytometry

After confirming isolation of EVs from two *L. cremoris* NZ9000 expressing recombinant proteins (and control strain containing empty plasmid), we included two additional recombinant proteins in our study and varied their expression mode. In the first instance, two proteins were simultaneously expressed (fARS019, mFyn) in a single *L. cremoris* NZ9000 species using dual cassette plasmid p-fARS019-mFyn. In the second instance, fARS019 was secreted instead of displayed on the surface. In the third instance, red fluorescent protein was expressed using plasmid p-Cher. Subsequent analyses confirmed the presence of vesicular structures in the ultracentrifuged samples and verified that the proteins expressed in the producer bacteria were present in the same samples.

**Fig. 2:**
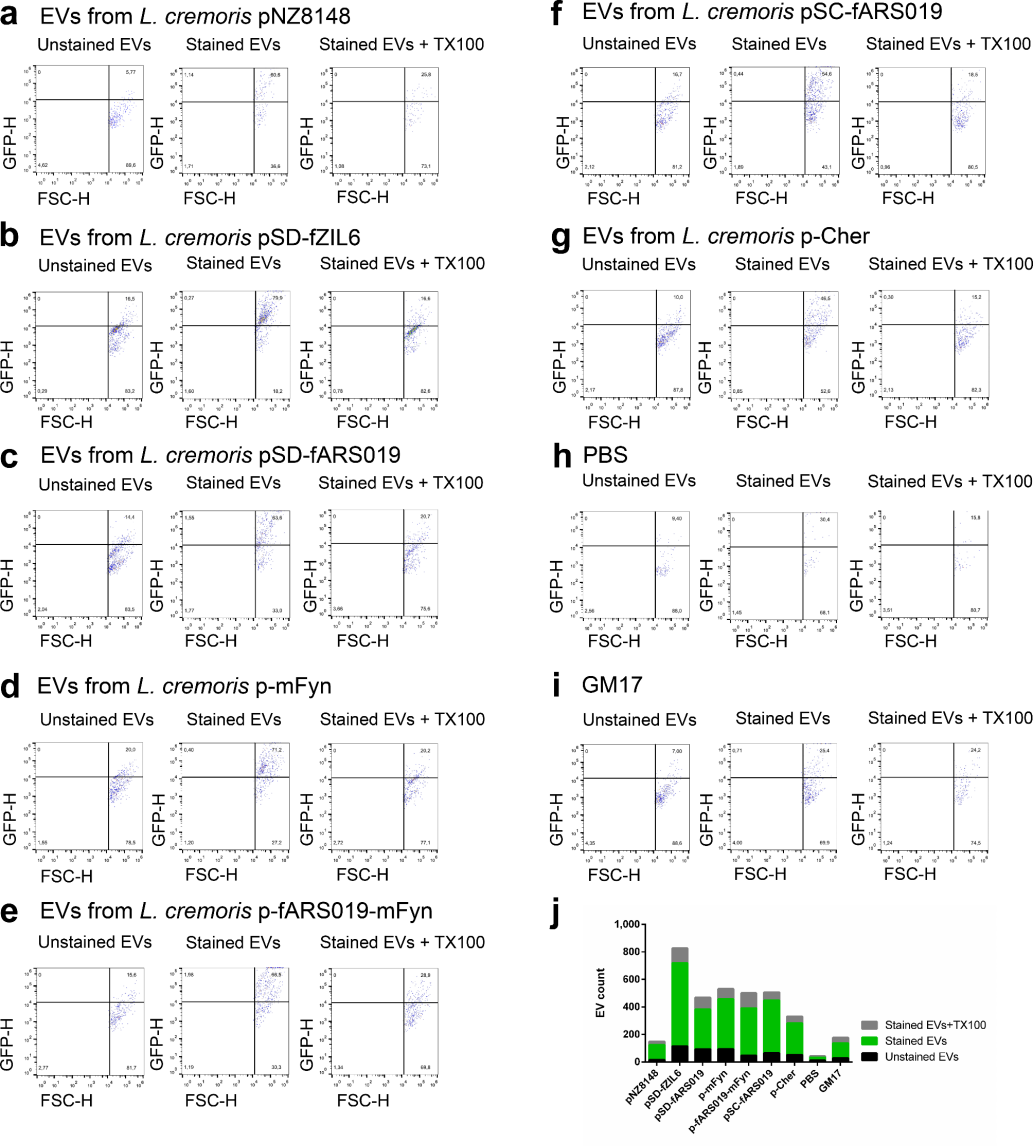
Flow cytometry analysis of the EVs from *L. cremoris* NZ9000 expressing recombinant proteins carrying plasmids pNZ8148 (**a**), pSD-fZIL6 (**b**), pSD-fARS019 (**c**), p-mFyn (**d**), p-fARS019-mFyn (**e**), pSC-fARS019 (**f**), p-Cher (**g**). Each sample was analyzed unstained, stained with the membrane dye, and treated with detergent TX100 after staining (**a**-**g**). Respective controls were included (**h**, buffer PBS and** i**, sterile bacterial growth medium). Scatterplots show the proportions (%) of EVs in each quadrant.** j**: Absolute number of green fluorescently labelled particles in the upper right quadrant.

To confirm the presence of the lipid bilayer membrane, the EVs were stained with the membrane-specific fluorescent dye FM1-43 and analyzed by flow cytometry. FM1-43 is a water-soluble dye, virtually non-fluorescent in aqueous solution, which becomes intensely fluorescent when incorporated into cytoplasmic membranes. An increase in fluorescence was observed in all stained EVs samples (Fig. 2), while considerably lower fluorescence signal was measured in their corresponding controls (i.e., unstained EVs, unstained and stained sterile growth media, unstained and stained resuspension buffer). This substantiated the presence of vesicular structures in isolated samples.

To confirm that the fluorescence resulted from incorporation of stain into the vesicular membrane, detergent Triton X-100 (TX100) was added to the stained samples. The detergent damages the bilayer membrane, and a decrease in fluorescence signal is expected. A drop in fluorescence signal was observed in all TX100-treated FM1-43-stained EVs samples, confirming the destruction of the lipid bilayer membrane (Fig. 2). Sterile growth medium that was not inoculated with bacterial cells was used as a control and processed in the same manner as cell free supernatants of cultures. Dot plots of the flow cytometry data and the gating strategy used to generate Fig. 2 are presented in Fig. S1.

**Fig. 3:**
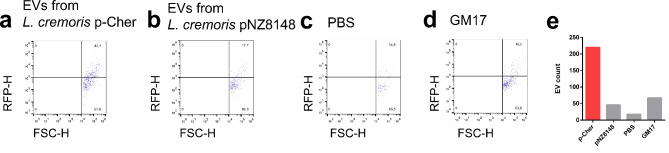
Flow cytometry analysis of the EVs from *L. cremoris* NZ9000 expressing red fluorescent protein mCherry. Respective controls were included (b, EVs from *L. cremoris* NZ9000 expressing proteins from plasmids pNZ8148, c, buffer PBS and d, sterile bacterial growth medium). Scatterplots show the proportions (%) of EVs in each quadrant. e: Absolute number of EVs in the upper right quadrant.

To detect the EVs, most studies use fluorescent labelling of the EVs, either with lipid dyes or fluorescent proteins^47–49^. To avoid the need of processing (staining) the EVs after their isolation, we used red fluorescent protein-producing strain (*L. cremoris* NZ9000/p-Cher) to isolate the EVs. EVs isolated from *L. cremoris* NZ9000/p-Cher exhibited red fluorescence in higher proportion than control samples (Fig. 3).

### Proteomic analysis

**Fig. 4:**
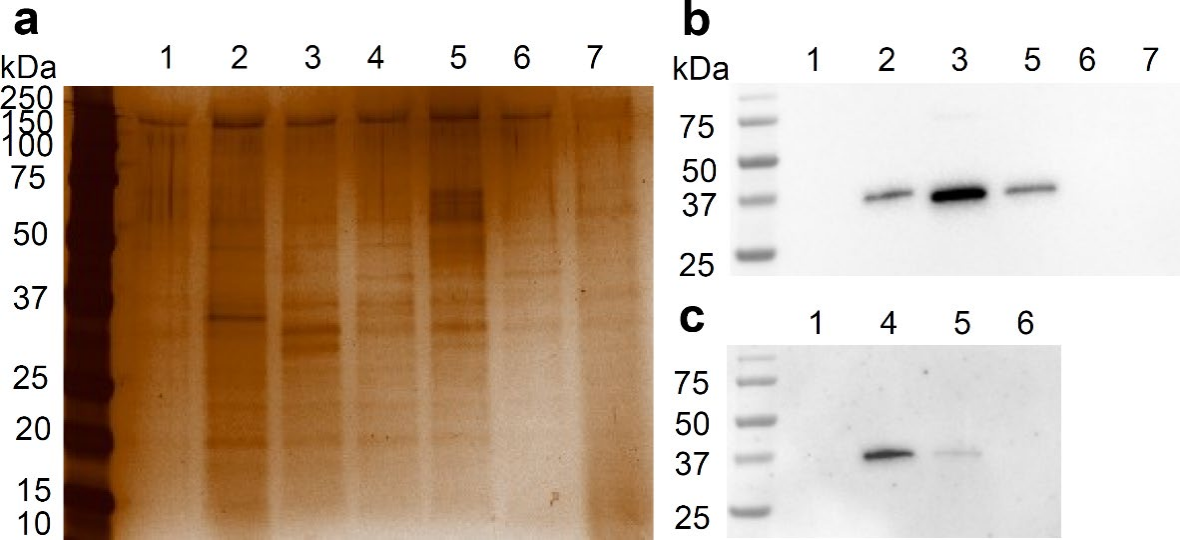
Proteomic analysis of the EVs with SDS-PAGE and silver staining (**a**) or representative images of Western blot (**b**,**c**). Full-length Western blots are included in Supplementary data as Fig. S3a and b. EVs containing proteins from *L. cremoris* NZ9000/pNZ8148 (1), pSD-fZIL6 (2), pSD-fARS019 (3), p-mFyn (4), p-fARS019-mFyn (5), p-Cher (6), pSC-fARS019 (7) were analyzed using anti-flag antibodies (b) or anti-myc antibodies (c). f represents inclusion of FLAG tag and m represents myc tag.

A preliminary characterization of the protein content of EVs-containing samples from recombinant *L. cremoris* NZ9000 was first performed by SDS-PAGE and silver staining. EVs-containing samples isolated from *L. cremoris* NZ9000 (Fig. 4a) and the corresponding whole protein samples obtained from the bacterial lysates (Fig. S2) was analyzed. Compared to the bacterial lysates, the protein content in EVs samples was lower. The EVs samples contained portion of the proteins detected in the bacterial lysates, and the major band corresponded in size to the recombinant protein expressed in *L. cremoris* NZ9000. Identity of the recombinant protein was confirmed with Western blot. Thus, the presence of the recombinant proteins expressed in *L. cremoris* NZ9000 in the EV-containing pellet after ultracentrifugation was confirmed (Fig. 4b and c and Fig. S3). We were able to confirm the presence of most recombinant proteins, with the exception of secreted fARS019 (possibly due to its small size), and mCherry (possibly due to lower expression level and lack of tag). For *L. cremoris* NZ9000 expressing two proteins simultaneously, we observed reduced expression of both proteins.

**Fig. 5:**
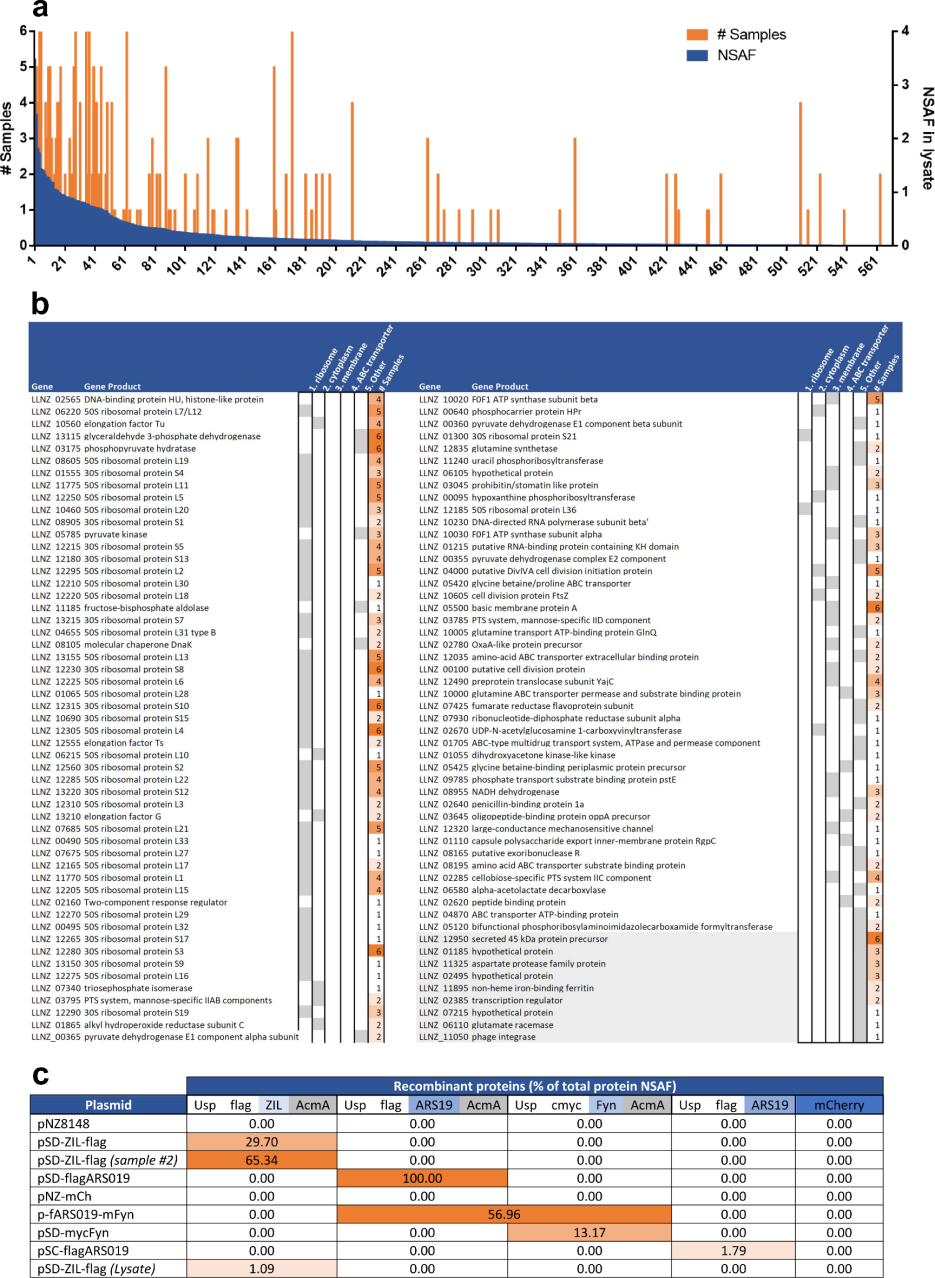
Mass spectrometry analysis of EVs from *L. cremoris* NZ9000. Individual proteins identified in EV samples were arranged according to their abundance in the cell lysate (determined as NSAF) and the number of EV samples in which they were identified is depicted (**a**). Gene and protein names of proteins identified in EV samples and their simplified genetic ontology. Of those, names of EV proteins that were not found in cell lysates are highlighted in gray (**b**). Occurrence of recombinant proteins in the EV samples and their percentage of total protein NSAF (**c**).

Mass spectrometry analyses of samples containing EVs identified 106 distinct *L. cremoris* NZ9000 proteins, of which 9 were not detected in the cell lysate, including the most abundant secreted protein Usp45 (Supporting data). Comparison of *L. cremoris* NZ9000 EVs-containing samples’ proteome with that of *L. cremoris* NZ9000 lysate revealed that proteins that were the most frequently found in EVs samples mostly correspond to the proteins that were the most abundant in the *L. cremoris* NZ9000 cell lysate according to the NSAF score (Fig. 5a). Among those proteins, various ribosomal proteins were by far the most frequent, followed by some cytoplasmic metabolic enzymes (glyceraldehyde 3-phosphate dehydrogenase, phosphopyruvate hydratase). Proteins that were also frequently present in the vesicles, but less abundant in the lysate, include membrane proteins and their subgroup, ABC transporters (Fig. 5b). All recombinant proteins, with the exception of protein mCherry, were detected in EVs samples. Portions of recombinant proteins’ NSAF in total protein NSAF depended on the number of distinct proteins identified in the sample, which differed between samples. This limited direct comparison of samples. Still, we found portions of total protein NSAF for recombinant proteins to be the lowest in the total cell lysate (1.09%) and in vesicles obtained from cells that secreted recombinant protein (below 1.79%). Vesicles obtained from cells that expressed and anchored recombinant proteins to their surface contained larger portions of total protein NSAF of recombinant proteins (13.17 − 100.0%) (Fig. 5c).

## Discussion

EVs are nanosized lipid bilayer particles released by different cellular organisms. While extensive research has been conducted on EVs derived from mammalian cells, the isolation of EVs from Gram-positive bacteria, especially probiotic Gram-positive bacteria, is relatively new. Compared to Gram-negative bacteria, vesicle production by Gram-positive bacteria has been overlooked due to the assumption that thicker cell wall structure and the absence of an outer membrane in Gram-positive bacteria affect the release of EVs. Nevertheless, this area shows great promise, especially in studying the anti-inflammatory effects of EVs released from probiotic lactic acid bacteria^33,50^.

The model lactic acid bacteria *Lactococcus lactis* and *L. cremoris* are well-characterized, and various genetic tools for their modification are available, which enable the expression of proteins with diverse effects, including anti-inflammatory^34,51^ and anti-cancer properties^52,53^. EVs from lactic acid bacteria also retain the probiotic beneficial effects^33,54^, suggesting that they have promising potential as alternative delivery vehicles, offering the same benefits as the originating bacteria while being smaller in size and capable of reaching distant targeting tissues. Most studies on EVs from lactic acid bacteria have focused on unmodified lactic acid bacteria. In contrast, this study aimed to analyze the formation of EVs by recombinant *L. cremoris* NZ9000 and the incorporation of bacterially expressed proteins into the EVs. We hypothesized that the over-expression of the recombinant protein could result in its spontaneous incorporation into EVs that will be present in the conditioned growth medium.

We assessed the feasibility of established *L. cremoris* NZ9000 system^39^ for recombinant protein expression to generate EVs. Recombinant proteins were expressed using a strong nisin-inducible promoter. EVs were isolated with ultracentrifugation, which is the gold standard for the EVs isolation from different sources, including the bacterial cells^25,33,54^. We expressed four different recombinant proteins in *L. cremoris* NZ9000 in different forms (fused with secretion signal or surface anchor). To explore the possibility of forming more complex EVs, we employed our recently developed BglBrick system for simultaneous expression of two proteins^40^. We confirmed that recombinant proteins were present in EVs-containing samples in considerable amount (13.17 − 100.00% of total protein content).

TEM imaging confirmed presence of cup-shaped vesicular structures with typical size of 50–200 nm, reported for bacterial EVs^55,56^, in three tested samples from *L. cremoris* NZ9000. When comparing the samples, EVs from *L. cremoris* NZ9000/pNZ8148 (empty plasmid control) exhibited greater diversity in size and were larger than those from *L. cremoris* NZ9000/pSD-fZIL6 and pSD-fARS019 (expressing recombinant proteins ZIL-6 and ARS019 in fusion with surface anchor, respectively). This observation was further supported by measurements of the polydispersity index, which demonstrated that the size of the produced EVs could be influenced by the expression of recombinant proteins in *L. cremoris* NZ9000, their secretion and surface anchoring. The zeta potential of the EVs was close to zero, indicating a tendency to aggregate, and suggesting low stability. In the literature, the measured zeta potential for exosomes from human cells shows a wide range, from − 10 mV to 50 mV, and can change quickly with variations in pH^57^, enzyme treatment^58^, and buffer composition^59^. For bacterial EVs, the ranges are also broad: from − 8.4 mV to -24.8 mV for EVs from *Pseudomonas aeruginosa*^60^, and more negative (− 54.3 mV and − 36.7 mV) for EVs from Gram-positive *Bacillus subtilis*^61^. It has been shown that the zeta potential of EVs is greatly influenced by the bacteria from which they originate^49^. The stability of our isolated EVs could potentially be improved by optimizing buffer composition and pH values^59^. Additionally, stabilizers such as trehalose and/or human albumin, which have previously been found to protect against aggregation^62–65^, could be added.

After initial confirmation of the presence of EVs in cell free supernatant of culture of recombinant *L. cremoris* NZ9000, we included additional recombinant proteins and analytical techniques in our study. The presence of EVs in the cell free supernatant of culture was confirmed by flow cytometry, using a membrane-specific fluorescent dye. The highest number of EVs was observed in samples of bacteria expressing recombinant proteins, especially those containing an anchoring domain, while the number of vesicles from culture of *L. cremoris* NZ9000 carrying empty plasmid was comparable to that of sterile growth medium. This suggests an important role of recombinant protein overexpression in vesicle formation, which has to be systematically evaluated in further studies. Moreover, the results suggest that specific protein features, such as presence of anchoring domains, are key factors in promoting inclusion of protein in EVs. Future studies will therefore focus on investigating these mechanisms in depth. Flow cytometry also confirmed the presence of red fluorescent signal in EVs from *L. cremoris* NZ9000/pCher expressing mCherry protein, while the signal was considerably lower in the respective controls. This finding supports the incorporation of recombinant proteins in EVs and underscores the potential future utility of recombinant fluorescent proteins for visualizing EVs as an alternative to fluorescent dyes, as well as confirming the inclusion of recombinant proteins into the vesicles.

To further confirm the presence of recombinant proteins, expressed by bacteria, in the EVs-containing samples, we employed two proteomic methods. First, presence of recombinant proteins of correct MW in the EVs samples was confirmed by SDS-PAGE and Western blotting using specific antibodies. Comparing the protein profile of *L. cremoris* NZ9000 lysates to that of EVs samples from *L. cremoris* NZ9000, we observed similarities, with lower amounts of proteins in the EVs. Recombinant proteins were detected with Western blot in all tested EVs-containing samples, except for the samples from *L. cremoris* NZ9000/pSC-fARS019 and *L. cremoris* NZ9000/p-Cher. EVs samples from *L. cremoris* NZ9000/pSC-fARS019 contained secreted version of the protein, which is problematic for detection due to its small size, as observed in our previous work^66^. Fluorescent protein mCherry was not fused with immune tag and, therefore, could not be detected with Western blot with antibodies against the tag. In the EVs samples from *L. cremoris* NZ9000 co-expressing two proteins, both could be detected, albeit at lower abundance than the recombinant proteins in the other samples, or when expressed separately. This is in agreement with previous observations of protein co-expression^30^. Nevertheless, both proteins were detectable, a finding further confirmed by mass spectrometry analysis.

Mass spectrometry analyses confirmed the presence of recombinant proteins within the EVs-containing samples, together with several intrinsic *L. cremoris* NZ9000 proteins. Among the latter, ribosomal proteins were by far the most abundantly detected. As these, together with some cytoplasmic metabolic enzymes, are also among the most abundant proteins in the soluble cell lysate, we hypothesize that EVs envelope parts of cytoplasm randomly, in non-regulated fashion. Membrane-associated proteins were also detected in EVs samples despite being less abundant in the cell lysate. This observation agrees with a relatively large membrane surface of the vesicles in comparison to that of the whole cells. Interestingly, basic membrane protein A that was among the most abundant surface exposed proteins in the previous study^67^, was also frequently detected in the vesicles; this again supports the non-regulated inclusion of proteins in the vesicles on the basis of their relative abundance. Presence of recombinant proteins, surprisingly with the exception of mCherry, was confirmed in all samples, corroborating the SDS PAGE and Western blot analyses. Recombinant proteins were mostly fusion proteins composed of multiple domains (Table 1), of which some were present in multiple fusion proteins (Usp, flag, cAcmA). This prevented quantitative separation of two concomitantly expressed proteins in vesicles obtained from cells transformed with p-fARS019-mFyn, as both fusion proteins contained cAcmA. In general, recombinant fusion proteins containing cAcmA (surface anchoring domain) were present in larger portions of total protein NSAF, which may be due to the stress caused by the effect of cAcmA, or due to the co-identification of intrinsic AcmA. The cAcmA and intrinsic AcmA could not be differentiated; however, intrinsic AcmA was not detected in control samples containing empty plasmid, suggesting majority of cAcmA peptides belong to recombinant fusion proteins.

## Conclusion

In this study we have confirmed that *L. cremoris* NZ9000 genetically engineered to express recombinant proteins produce EVs. Our data also support the presence of recombinant proteins in the EVs, as they were detected in the EVs present in ultracentrifugation pellets. However, additional experiments will be needed to confirm the actual incorporation of recombinant proteins other than mCherry in EVs (e.g. immuno-TEM). Recent studies have shown that EVs carrying recombinant proteins can be utilized in biotechnological applications, primarily focusing on the production of vaccines and eliciting sufficient immune responses^55,68^, as well as the development of high-yield vesicle-packaged proteins^69^. However, these studies have primarily focused on EVs from Gram-negative *E. coli*. As *L. cremoris* NZ9000 is considered safe bacterium from the group of LAB, EVs from *L. cremoris* NZ9000 could have several advantages over EVs from other bacteria. Our findings thus provide a scalable method for production of EVs with potential for efficiently incorporating recombinant proteins. The results of this study indicate that the anchor domain on proteins may be crucial for guiding recombinant proteins into EVs and enabling them to become high-yield vesicle-packaged proteins, which will be the subject of future research in which recombinant proteins with and without the anchor domain will be expressed and their quantitative presence in EVs analyzed. In the future, such EVs could potentially be considered as alternative delivery vehicles for recombinant therapeutic proteins.

## Electronic supplementary material

Below is the link to the electronic supplementary material.

Supplementary Material 1

Supplementary Material 2

## Data Availability

The data and materials used in this study are available from the corresponding author upon request.
