# Supplementary material for: Incorporation of recombinant proteins into extracellular vesicles by Lactococcus cremoris
Source: Sci Rep. 2025 Jan 13;15:1768. doi: 10.1038/s41598-025-86492-z (PMC11736121; doi:10.1038/s41598-025-86492-z)
Supplement: Supplementary file 1 — Supplementary Material 1 [file 41598_2025_86492_MOESM1_ESM.pdf]

Supplementary data

Incorporation of recombinant proteins into extracellular vesicles by *Lactococcus cremoris*

Tina Vida Plavec [1,4], Kristina Žagar Soderžnik [2], Giulia Della Pelle [2], Špela Zupančič [4], Robert Vidmar [3], Aleš Berlec [1,4,*]

[1] Department of Biotechnology, Jožef Stefan Institute, Ljubljana, Slovenia
[2] Department for Nanostructured Materials, Jožef Stefan Institute, Ljubljana, Slovenia
[3] Department of Biochemistry and Molecular and Structural Biology, Jožef Stefan Institute, Ljubljana, Slovenia
[4] University of Ljubljana, Faculty of Pharmacy, Ljubljana, Slovenia

* Correspondence: ales.berlec@ijs.si

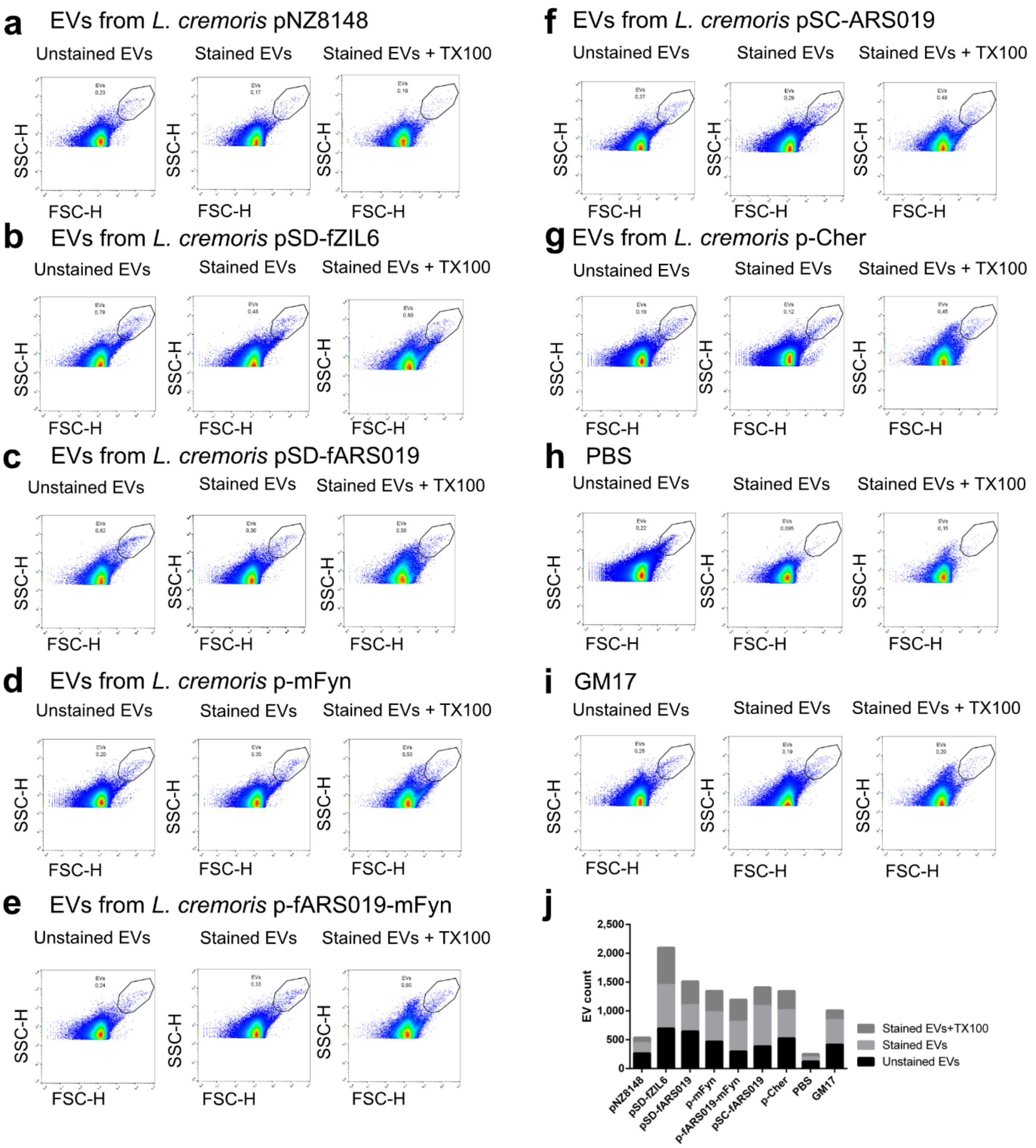

Fig. S1. Flow cytometry analysis of distribution of EVs from *L. cremoris* NZ9000 expressing different recombinant proteins expressing recombinant proteins pNZ8148 (a), pSD-fZIL6 (b), pSD-fARS019 (c), p-mFyn (d), p-fARS019-mFyn (e), pSC-fARS019 (f), p-Cher (g), showing unstained samples, samples stained with the membrane dye, and detergent TX100 treated stained samples (a-g) in the dot plot display mode of forward scatter (FSC) versus side scatter (SSC). Respective controls were included (h, buffer PBS and i, bacteria growth medium). Number of EVs in the gated field (j).

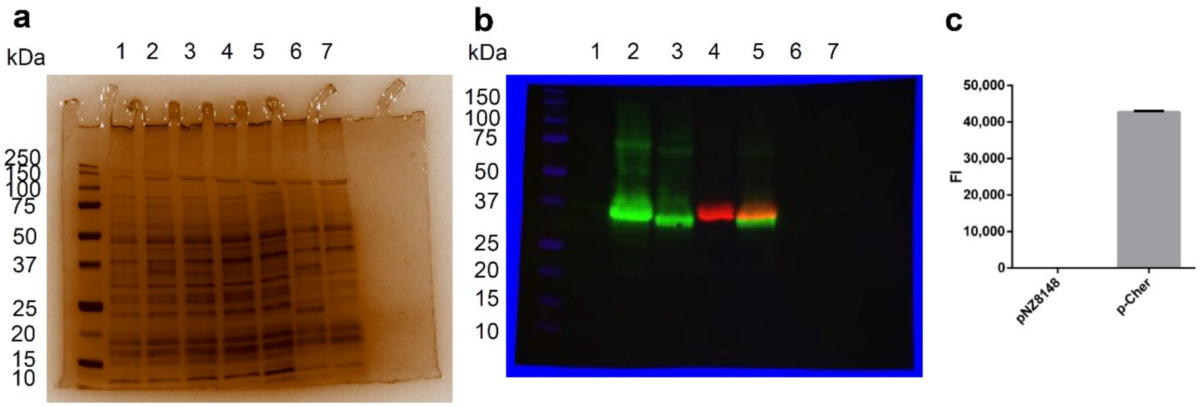

Fig. S2. Original photos of proteomic analysis of the *L. cremoris* NZ9000 lysates by SDS-PAGE and silver staining (a) or Western blot (b). *L. cremoris* NZ9000/pNZ8148 (1), pSD-fZIL6 (2), pSD-fARS019 (3), p-mFyn (4), p-fARS019-mFyn (5), p-Cher (6), pSC-fARS019 (7) were analyzed using anti-flag antibodies or anti-myc antibodies. f represents inclusion of FLAG tag and m represents myc tag. Fluorescence intensity measurement of *L. cremoris* NZ9000/p-Cher (c).

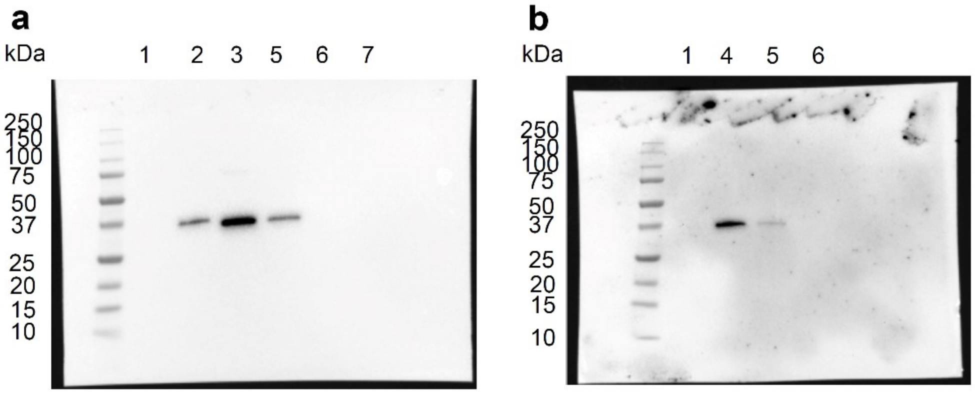

Fig. S3. Original photos of proteomic analysis of the EVs with SDS-PAGE and Western blot. EVs containing proteins from *L. cremoris* NZ9000/pNZ8148 (1), pSD-fZIL6 (2), pSD-fARS019 (3), p-mFyn (4), p-fARS019-mFyn (5), p-Cher (6), pSC-fARS019 (7) were analyzed using anti-flag antibodies (a) or anti-myc antibodies (b). f represents inclusion of FLAG tag and m represents myc tag.
